# Measuring Multidimensional Health Poverty in China

**DOI:** 10.3389/fpubh.2021.786325

**Published:** 2022-01-31

**Authors:** Xin-Xin Chi, Xi-Hua Liu, Ze-Zhong Zhang

**Affiliations:** ^1^School of Economics, Qingdao University, Qingdao, China; ^2^School of Economics and Management, Qinghai Minzu University, Xining, China

**Keywords:** multidimensional health, health poverty, income poverty, CHE, China

## Abstract

This article defines the concept of “multidimensional health poverty,” considering both the monetary aspects and multidimensional health deprivation of health poverty. Moreover, we set up the multidimensional health poverty index (MHPI) to measure health poverty in China by revising the traditional A-F MPI method, specifically we use the Catastrophic Health Expenditure (CHE) as a sufficient condition and income poverty as a necessary condition, and take physical, mental, and social health into account. The measurement result evidences that physical health, monetary dimensions (CHE and income poverty), and mental health contribute most to health poverty in China. In addition, the MHPI is significantly higher in rural areas than urban because of higher out-of-pocket medical payments and health deprivation in more dimensions. Compared with the traditional method, the MHPI is more accurate, stable, and comprehensive, making it more suitable for measuring health poverty.

## Introduction

According to the sustainable development goals (SDGs), we expect to end poverty in all its forms everywhere, ensure healthy lives, and promote wellbeing for all ages up to 2030 (1). However, with the recent coronavirus disease 2019 (COVID-19) outbreak, to realize these two goals is full of uncertainty and difficulties (2, 3). Progress toward the two goals is closely linked and highly aligned (4, 5). There is a large literature on the relationship between health and poverty (3–5). Health is also an important and commonly used dimension when measuring multidimensional poverty (2, 6). However, unlike the notion of multidimensional poverty, health poverty is rarely used and measured by itself, although not entirely new (7). As a result, it is difficult to identify who needs help because of the poverty caused by health. Therefore, by measuring health poverty precisely and roundly, this study can benefit the decision-makers to focus on those who need help because of poverty caused by health issues and develop sound public health policies.

Among the governments worldwide, the Chinese Ministry of Health first put forward the concept of health poverty and took the health poverty alleviation project as an important part of the “Targeted Poverty Alleviation” practice. The Chinese government attaches great importance to health poverty alleviation and has accumulated a large amount of practical experience. Therefore, it is representative to use Chinese data to study health poverty. Although China has eliminated absolute poverty by 2020, as the most populous country globally, the problem of returning to poverty is still serious. Given that poverty due to illness is the main cause of multidimensional poverty in China, accounting for 44%, and the impact of COVID-19, health poverty is still the primary focus in the post-poverty era (8–10). As for health poverty, previous studies and government policies in China have focused on monetary poverty caused by illness, that is, the huge medical expenditure and income loss caused by illness, and lack of attention to health capacity deprivation and multidimensional health (11–14). To prevent returning to poverty due to illness and promote health equity, the government should pay adequate attention to the monetary and non-monetary aspects of health. In addition to China, many countries in the world also face the problem of health poverty. Studies have found that health poverty is a key contributor to multidimensional poverty in India and the richer countries of Europe (15, 16). Therefore, more comprehensive and targeted research on health poverty is necessary.

Scholars have not yet agreed on the definition of health poverty. To sum up, health poverty is mainly defined from the following two perspectives: First, in the vision of poverty due to illness, health poverty is defined as monetary poverty caused by health problems, mainly including poverty caused by huge medical expenditure and income reduction due to declining health levels (17–19). This literature often measures health poverty by Catastrophic Health Expenditure (CHE) or Impoverishment Health Expenditure (IHE), ignoring groups that do not suffer from CHE or IHE but have low income due to health problems. Second, based on the theory of human capital and feasible ability theory, health poverty is defined as the lack of individual health capital and the deprivation of health ability (7, 20). Health indicators used are relatively simple, ignoring the multidimensional nature of health. People with poor health measured in this way may have enough money to receive adequate medical support and do not require government help. Very little literature defines health poverty as a state where health deprivation and low income coexist but has not measured it empirically (21–23). This literature revises the traditional A-F MPI method to construct a new index measuring multidimensional health poverty and applies the 2018 wave of the China Family Panel Studies to conduct an empirical analysis. The new index considers both the monetary and multidimensional health deprivation and performs more accurately, stably, and comprehensively than other health poverty measurement methods, and is more suitable for measuring health poverty. In addition, the new index can be flexibly adjusted to measure multidimensional health poverty in other countries, filling in gaps in these studies. We further apply a decomposition analysis on the multidimensional health poverty index to understand the contribution of each indicator to multidimensional health poverty and the differences between urban and rural multidimensional health poverty in China. These analyses suggest that the government should further reduce the medical expenditure burden of rural households and pay attention to multidimensional health.

The rest of the paper is structured as follows: Section Literature Review reviews the existing literature, whereas Section Intuitive Explanation of the Construction of Multidimensional Health Poverty Index intuitively introduces the multidimensional health poverty index (MHPI). Section Methodology presents the construction method of MHPI. Section Data, the Selection of Dimension, Cutoff, and Weight describes the data, selection of dimension, cutoff, and weight. Section Measurement Outcomes is the measurement outcomes. Section Discussion and Conclusion offers discussions and conclusions.

## Literature Review

When it comes to measuring health poverty, most studies consider low health levels as health poverty from the perspective of health deprivation (7, 24). The most commonly used method is self-reported health (SRH), in which individuals are asked to rate their health in five or so grades. Bennett and Hatzimasoura (25) used ordinal self-reported data on health status to examine health poverty in Canada and the United States. Brzezinski (26) estimated trends of health poverty in Britain using ordinal SRH, and Pascual-Saez et al. (27) did the same for Spain.

However, Simões et al. (24) argued that the SRH index is influenced by inaccuracies stemming from reporting heterogeneity. Alternatively, the authors consider the EuroQol (EQ-5D) index to construct a quasi-objective health indicator, which defines health in terms of five dimensions: mobility, self-care, usual activities, pain/discomfort, and anxiety/depression. Similarly, Clarke and Erreygersa (7) measured health poverty with three health indicators: cardiovascular risk, life expectancy, and the SF-6D, namely, six multilevel dimensions: physical functioning, role limitations, social functioning, pain, mental health, and vitality. One is considered health poor when his or her health falls below minimally acceptable thresholds. Based on the fuzzy set theory, Alperin (28) constructed the multidimensional health index (MHI), exploiting nine items that reflect individual mental and physical health status. Aurino and Burchi (29) construct the multidimensional health poverty index (MHPI) to measure early childhood health deprivation, and the index is composed of Height-for-Age Z-score (HAZ), Weight-for-Height Z-score (WHZ), and life-threatening diseases with equal weights. Bai and Gu (30) construct a multidimensional health deficiency duration index to investigate health trends for the elderly; the index is composed of Activities of Daily Living (ADLs), Instrumental Activities of Daily Living (IADLs), cognitive functions, SRH, and negative emotions.

The above studies embody the idea that health is multidimensional and use the multi-attribute index to measure health poverty. Nevertheless, most of the literature on health is focused on physical and mental aspects (26), ignoring social health. The WHO defines health as a state of complete physical, mental, and social wellbeing and not merely the absence of disease or infirmity (31). However, few studies have taken social wellbeing as a health dimension to measure multidimensional health. Social health refers to the interaction between the individual and the society in the social role, including personality and social skills (32, 33). Some scholars believe that social health includes social adaptation and social support (34). In a word, social health reflects the sense of gain and identity of an individual from social activities, and is an organic component of multidimensional health. It interacts with physical and mental health and affects the use by an individual of social resources, thus affecting the economic status of the individual (35, 36). Therefore, to make a more comprehensive evaluation of multidimensional health, this study considers physical and mental health and social health.

Moreover, the above studies on health poverty are focused on health status while neglecting the monetary aspects of health poverty. Based on the capability theory of health, we can measure out the multidimensional health status of people, but it could not help the government screen out the health poverty population in need of help. That is because individuals with poor health status may not need to pay large medical expenses or have high income and do not need financial support from the government. According to Grossman's human capital theory, health is an investment product, and low health levels will lead to underinvestment in human capital and increase healthcare spending, leading to poverty (37). Therefore, this study takes household income and medical expenditure into account to measure multidimensional health poverty.

Firstly, poor health leads to low income (37). However, there are households in which some members are in poor health but the others have high incomes. Such households can afford treatment and adequate high-quality food without worrying too much about the loss of household income due to poor health. Therefore, when measuring poverty on a household basis, the co-existence of poor health status and income poverty is a key indicator of health poverty (21–23). In this study, we regard income poverty as a necessary condition, that is, when a household is in poor health but not in income poverty, we identify that it is not in multidimensional health poverty.

Besides, if we measure health poverty solely based on income and health status, we will ignore those who are not relatively poor but face significant out-of-pocket medical payments. As early as 2001, the World Bank has acknowledged that out-of-pocket payments for health services, especially hospital care, can make the difference between a household being poor or not (38). Sterck et al. (39) derived a health poverty line based on basic health care costs and health spending at 5% of income. Antosova et al. (40) constructed a health poverty index to show how much money households lack to meet prescribed standards of health care. Beyond that, CHE is a more commonly used and widely accepted indicator in the international health economy to measure the economic risk of poor health, which is defined as out-of-pocket health care expenditure that exceeds a certain percentage of household income and imposes a disease burden on the household (11, 41, 42). Once CHE occurs, households are immediately plunged into a state of health poverty and need social assistance. Hence, we take CHE as a sufficient condition of health poverty; in other words, if a household suffers CHE, we consider it to be in multidimensional health poverty, regardless of other indicators.

When measuring health poverty in a single or composite item, the Foster-Greer-Thorbecke (FGT) index is often adopted (7, 23–25). Apart from that, Alperin (28) constructed the MHI based on the fuzzy approach proposed by Cerioli and Zani (43). As for more dimensions, Aurino and Burchi (29) adopt the Alkire-Foster method (6), which extends the FGT index from one dimension to more dimensions to measure childhood health. Bai and Gu (30) constructed a multidimensional health deficiency duration index based on the Alkire-Foster method (6) and the duration approach of Foster (44). Furthermore, taking income poverty as a necessary condition of multidimensional poverty, Zhang et al. (45) built the income-oriented multidimensional poverty index. We improve on the approach of Zhang by making income poverty a necessary condition and CHE a sufficient condition, and taking physical, mental, and social health into account.

## Intuitive Explanation of the Construction of Multidimensional Health Poverty Index (MHPI)

Before revising the Alkire-Foster MPI method, we first intuitively explain the difference between MHPI and traditional Alkire-Foster MPI. Here, the MHPI is defined as follows: When an individual (here the “individual” can be a person or a household) is deprived in the CHE dimension, the individual is at least in one-dimensional health poverty. Moreover, if the individual is deprived of income and a certain aspect of health, the individual is at least in two-dimensional health poverty. Otherwise, if the individual is only deprived in income dimension or not deprived in any dimension, then the individual is not in health poverty. By contrast, in Alkire-Foster's perspective of multidimensional poverty, the attributes of each dimension are the same (although the weights of different dimensions can be different). As long as any dimension is deprived, the individual is identified as poor in at least one dimension.

Take Table 1 as an example. The deprivation matrix is a multidimensional health poverty deprivation matrix of eight individuals. Each row represents individual i, each column represents a dimension j, the first column is the CHE dimension, and the second column is the income dimension. The element meaning of the matrix is as follows: 1 represents that the individual is deprived in that dimension, and 0 means that there is no deprivation. Table 2 points out the results of different identification indexes. From the perspective of MHPI, individuals 1, 3, 4, and 8 are at least in one-dimensional poverty, because they are all deprived of CHE. As the *k* increases, individuals 1, 3, 4, 5, 6, 7, and 8 are at least in two-dimensional poverty. Individual 2 is never considered as health poor, because he or she is only deprived of income. In the multidimensional poverty perspective of Alkire and Foster, eight individuals are at least one-dimensional health poor.

**Table 1:** Deprivation matrix.

** 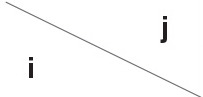 **	**1**	**2**	**3**	**4**	**5**
1	1	0	0	0	0
2	0	1	0	0	0
3	1	1	0	0	0
4	1	0	1	0	0
5	0	1	1	0	0
6	0	1	1	1	0
7	0	1	1	1	1
8	1	1	1	1	1

**Table 2:** Identification of multidimensional health poverty under different indexes.

**Dimension**	**Health poverty defined by MHPI**	**Health poverty defined by conditional A–F**
*k* = 1	1, 3, 4, 8	1, 2, 3, 4, 5, 6, 7, 8
*k* = 2	1, 3, 4, 5, 6, 7, 8	3, 4, 5, 6, 7, 8
*k* = 3	1, 3, 4, 6, 7, 8	6, 7, 8
*k* = 4	1, 3, 4, 7, 8	7, 8
*k* = 5	1, 3, 4, 8	8

From the above comparison, we can see the difference between MHPI and A–F MPI: MHPI focuses more on the multidimensional characteristics of individuals who are deprived in the income or expenditure dimension, which can measure out individuals in need of government assistance. Thus, health poverty alleviation policies can be targeted more precisely.

## Methodology

Like the A-F MPI, MHPI is built on the poverty deprivation matrix. In particular, let *y* = |*y*_*ij*_| denote the *n* × *d* matrix, where *n* is the total number of individuals (which can be a person or household) to be investigated, and *d* is the dimension of poverty. Then, *y*_*ij*_denotes individual*i's* achievement in dimension *j*. Let *z*_*j*_ > 0 denote the cutoff, below which a person is considered to be deprived in dimension *j*.

First, calculate the deprivation matrix $g^{0}=|g_{ij}^{0}|$, whose typical element $g_{ij}^{0}$ is defined by $g_{ij}^{0}=1$ when *y*_*ij*_ < *z*_*j*_, while *g*_*ij*_ = 0 otherwise. Each row vector *g*_*i*_ lists individual *i*′*s* deprivation vector.

Second, determine the health poverty deprivation count function that contains the CHE and income dimension. From the matrix *g*^0^, we denote $c_{i}=|g_{i}^{0}|$, which represents the number of deprivation suffered by individual *i*. Assume that the first column of the deprivation matrix is the CHE dimension and that the second column is income where *E* is the deprivation cutoff value of the CHE dimension, and *I* is the deprivation cutoff value of the income dimension. When there is no deprivation in the CHE dimension and no deprivation in the income dimension, or there is deprivation only in the income dimension, the deprivation count *c*_*i*_ equals 0.

Finally, determine whether an individual is health poor by the health poverty cutoff. With the health poverty cutoff *k*, define the identification function ρ_*k*_(*y*_*i*_ ; *z*) = 1 when there is deprivation in CHE or *c*_*i*_ ≥ *k* and ρ_*k*_(*y*_*i*_ ; *z*) = 0 whenever *c*_*i*_ < *k*. ρ_*k*_(*y*_*i*_ ; *z*) = 1 means that individual *i* is at least deprived in *k* dimensions and identified as health poor. After identifying health-poor individuals, we can add up the total number of health-poor individuals in the sample and then calculate the incidence of multidimensional health poverty *H* as follows:


(1) $$\begin{array}{l} H=q/n \end{array}$$

where *q* is the total number of individuals identified as health poor; moreover, we define the average deprivation share *A*, which shows the average share of deprivation among the health poor:


(2) $$\begin{array}{l} A=\frac{1}{q}\overset{q}{\underset{i=1}{\sum }}c_{i}\left(k\right) \end{array}$$

Thus, we can calculate the multidimensional health poverty index *M*_0_:


(3) $$\begin{array}{l} M_{0}=H\cdot A=\frac{1}{n}\overset{n}{\underset{i=1}{\sum }}c_{i}\left(k\right) \end{array}$$

The MHPI constructed with the revised A-F method is similar to the MPI constructed with the A-F method but has different meanings. The difference is that the value of ρ_*k*_(*y*_*i*_ ; *z*) recognition function adjusts the structure of MPI, so that the MHPI under different cutoff *k* always takes monetary factors into account.

*M*_0_ has a key property named “decomposability,” which can break down the overall poverty into a weighted average of subgroup poverty levels, where the weight is the population share of the subgroup (6). Divide the entire population into two mutually exclusive groups *y*_*a*_ and *y*_*b*_. Denote by*n*(*y*_*a*_*)* the number of individuals in*y*_*a*_and similarly for *n*(*y*_*b*_) and *n*(*y*_*a*_, *y*_*b*_). Then, the multidimensional health poverty index *M*_0_ can be expressed as


(4) $$\begin{array}{l} M_{0}\left(y_{a}\;,y_{b}\right)=\frac{n\left(y_{a}\right)}{n\left(y_{a}\;,y_{b}\right)}M\left(y_{a}\;;\;z\right)+\frac{n\left(y_{b}\right)}{n\left(y_{a\;},y_{b}\right)}M\left(y_{b}\;;\;z\right) \end{array}$$

By decomposing *M*_0_, we can understand the contribution rates of various subgroups to the overall health poverty and compare health poverty status among different groups.

## Data, the Selection of Dimension, Cutoff, and Weight

### Data

The dataset considered is the 2018 wave of the China Family Panel Studies (CFPS), covering 31 provinces, autonomous regions, and municipalities directly under the Central Government of China. By conducting a follow-up questionnaire survey of different families and individuals, it can reflect the economic, educational, and health status of the families living in China. The questionnaire is divided into three types: village (residential) questionnaire, household questionnaire, and individual questionnaire (46). We use the household questionnaire and the corresponding individual questionnaire in this study and merge household-level data with individual-level data. If any household-level information for health poverty measurement is missing, or the individual level information of all members in the household is missing, the household will be excluded. The total sample size remaining after our treatment is 10,455 households and 36,912 individuals.

### Dimensions and Cutoff

The underlying dimensions of multidimensional health poverty are extensive and include both monetary aspects and multidimensional health status. Table 3 shows dimensions, indicators, and cutoffs of the multidimensional health poverty index (MHPI). On the monetary front, we make CHE a sufficient condition and income a necessary condition; in terms of multidimensional health, we consider physical, mental, and social health. Considering the mutual aid and interaction within the household, measuring the individual health poverty solely is not complete (47, 48). Therefore, the household is regarded as the minimum identification unit. We use all the household member information to determine whether the household is in multidimensional health poverty or not. If the household has CHE or has no CHE but is in relative income poverty and has members with physical, mental, or social health problems, we identify the household as being in multidimensional health poverty.

**Table 3:** Dimensions, indicators, and cutoffs of the multidimensional health poverty index (MHPI).

**Dimension**	**Indicator**	**Deprived if**
Medical expenditure	CHE	The household has suffered CHE over the 12 month recall period
Income	Relative income poverty	The household's per capita disposable income falls below the relative income poverty line
Physical health	SRH	At least one household member reports poor self reported health status
	PADLs difficulties	At least one household member can't go out for outdoor activities, eat, perform kitchen activities, take public transportation, go shopping, do cleaning, or do laundry independently
	Chronic diseases	At least one household member has doctor-diagnosed chronic disease in past 6 months
	Nutrition	Any adult or child whose nutritional information is malnourished
Mental health	Depression	At least one household member has a depression score over 40
	Cognitive impairment	At least one household member has a cognitive score below 40% of the median
Social health	Interpersonal relationships	At least one household member has a popularity score below 5 points
	Social trust	At least one household member has a total score of trust <25

*Nutrition deprivation standard: children (5–19 years old) are regarded as malnourished if their body mass index (BMI) z-scores fall below the median of the reference population of the same age and gender minus two standard deviations; adults are regarded as malnourished with BMI < 18.5. The criterion is from WHO: https://www.who.int/toolkits/growth-reference-data-for-5to19-years/application-tools*.

For each health dimension, appropriate, and comprehensive indicators are selected based on previous research studies and data availability. For CHE, as in most of the literature, we calculate it performing the WHO method, defined as the out-of-pocket payment (OOP) for healthcare ≥40% of household income (49, 50). On the income dimension, given that China has completely eliminated absolute poverty, we use relative income poverty to measure income status. Developed countries mostly use 50–60% of the median income as the relative poverty line (51). As a developing country, China is still in the initial stage of relative poverty. If we use the relative poverty line of developed countries, it will create a large number of relatively poor groups. In addition, the long-term existence of the urban-rural dual economic structure has led to a large income gap between urban and rural residents in China. If the unified urban-rural relative income poverty line is adopted, the number of people living in relative poverty will be large and concentrated in rural areas. Therefore, based on the income distribution situation of China and relevant research (52), we calculate the relative poverty line at 40% of the median *per capita* disposable income for urban and rural areas.

According to the definition of health by the World Health Organization, we select health indicators from three dimensions of physical, mental, and social health (31). The physical health dimension comprises four indicators: SRH, Physical Activities of Daily Living (PADLs), chronic diseases, and BMI. SRH is a commonly used indicator in measuring health; it is complex and can be influenced by the adaptive preferences, mood, and personality type of a person (53). This makes it relate to other health indicators, but as a supplement of the other explicit health indicators, we suggest that using it mainly to detect those who believe that their health is poor and lack in the ability to work is reasonable. If there is someone in the household reporting that his or her health is poor, the household is considered deprived in the SRH dimension. Inversely, the household is considered non-deprived if none of them report poor SRH. The second physical health indicator is PADLs. If there are household members having difficulties in PADLs, they will need the care of others, which will inevitably lead to the decline in the ability of the whole household to work, therefore, poverty. Similarly, having chronic diseases may impair the ability of a person to work and cause medical costs. Finally, undernutrition can make a person vulnerable to diseases and have life-long effects on physical and cognitive development for children (54, 55). Meanwhile, it usually indicates that the household is in poverty (56). Therefore, we take the BMI as a physical health indicator. It is worth mentioning that, when measuring the nutritional status of a child, we follow the standards set by the WHO. Children older than 5 years old are regarded as malnourished if their BMI z-scores fall below the median of the reference population of the same age and gender minus two standard deviations. For children whose age is between 0 and 5, since birth month is not provided in the data, and the weight of children born in different months varies greatly, we exclude the nutritional status of children under 5 years old^1^

This study mainly adopts two kinds of mental health indicators: depression and cognitive impairment. Depression is the primary indicator of mental health evaluation. We identify a person to be in depression if his or her CESD20 scores are higher than 40. Cognitive ability is another indicator of mental health and is a form of health like physical health and depression. Impairment of individual health status will be clearly reflected in cognitive function, typical of which is a neurodegenerative disease. Some severe mental disorders damage cognitive function even more than physical function (57). The CFPS2018 survey uses a set of literacy questions and a set of mathematical questions to test and assess the cognitive levels of respondents, and we use the sum scores of the word test scores (0–34) and math test scores (0–24) to represent their cognitive health. Individuals who score below 40% of the median are considered to have cognitive health impairments.

For social health, restricted by data availability, this study uses two indicators: interpersonal relationships and social trust. Interpersonal relationships reflect the sense of identity gained by individuals in social relations. Good popularity often means that individuals can get more social care and support when they encounter difficulties. The CFPS2018 gives a self-reported popularity score from 0 to 10. Individuals with scores <5 are identified as deprived of interpersonal relationships. In addition, there is a significant relationship between social trust and individual health (33, 58). First, individuals with higher social trust are more likely to participate in social activities and, thus, have higher self-reported health. Second, social trust may have extrinsic effects, as trust at the individual level affects the sociopolitical environment and, therefore, affects the health status of an individual (34). In the CFPS2018 survey, social trust is divided into six categories according to objects: parents, neighbors, Americans, strangers, cadres, and doctors. According to the level of trust, the values of trust range from 0 to 10, where 0 means very distrusting, and 10 means very trusting. This study considers the level of trust of an individual for multiple related subjects and adds up the six indicators. Individuals who score <25 are considered lacking in social trust.

### Weighting

For ease of calculation, all dimensions are given equal weight here. As for the cutoff *k* of the multidimensional health poverty, its selection is different because of the difference of health poverty alleviation standard, poverty dimension selection, and other factors. Considering that the evaluation system has nine dimensions, we calculated the corresponding MHPI and investigated the difference in the measurement results.

## Measurement Outcomes

### The Measurement of Multidimensional Health Poverty

This study presents the results of multidimensional health poverty indexes under different thresholds. According to the calculation results shown in Table 4, the multidimensional health poverty reflected by different poverty cutoffs varies considerably. When *k* = 1, the results reflect the MHPI measured by CHE, the number of health poverty households identified is 1,043, and the incidence of health poverty is 9.98%; that is, 1,043 households are suffering CHE, accounting for 9.98% of all households. When *k* = 2, the measurement results reflect the expenditure health poverty measured by CHE, the income health poverty measured by relative income poverty, and one-dimensional non-monetary health poverty. As shown in Table 4, since including income health poverty, the MHPI increases rapidly when the poverty cutoff goes from 1 to 2 and then decreases gradually with the increase in non-monetary health dimensions.

**Table 4:** MHPI, *q, H*, and *A* in different cutoff *ks*.

***k***	***M_**0**_***	***q***	***H%***	***A***
1	0.0475	1,043	9.98	0.4763
2	0.0972	2,327	22.26	0.4366
3	0.0924	2,078	19.88	0.4649
4	0.0833	1,761	16.84	0.4946
5	0.0739	1,514	14.48	0.5100
6	0.0632	1,292	12.36	0.5118
7	0.0550	1,149	10.99	0.5008
8	0.0504	1,080	10.33	0.4881
9	0.0481	1,050	10.04	0.4791

*M_0_ is the multidimensional health poverty index, q is the number of health poverty household, H is the incidence of the health poverty, and A is the average deprivation score*.

**Figure 1:**
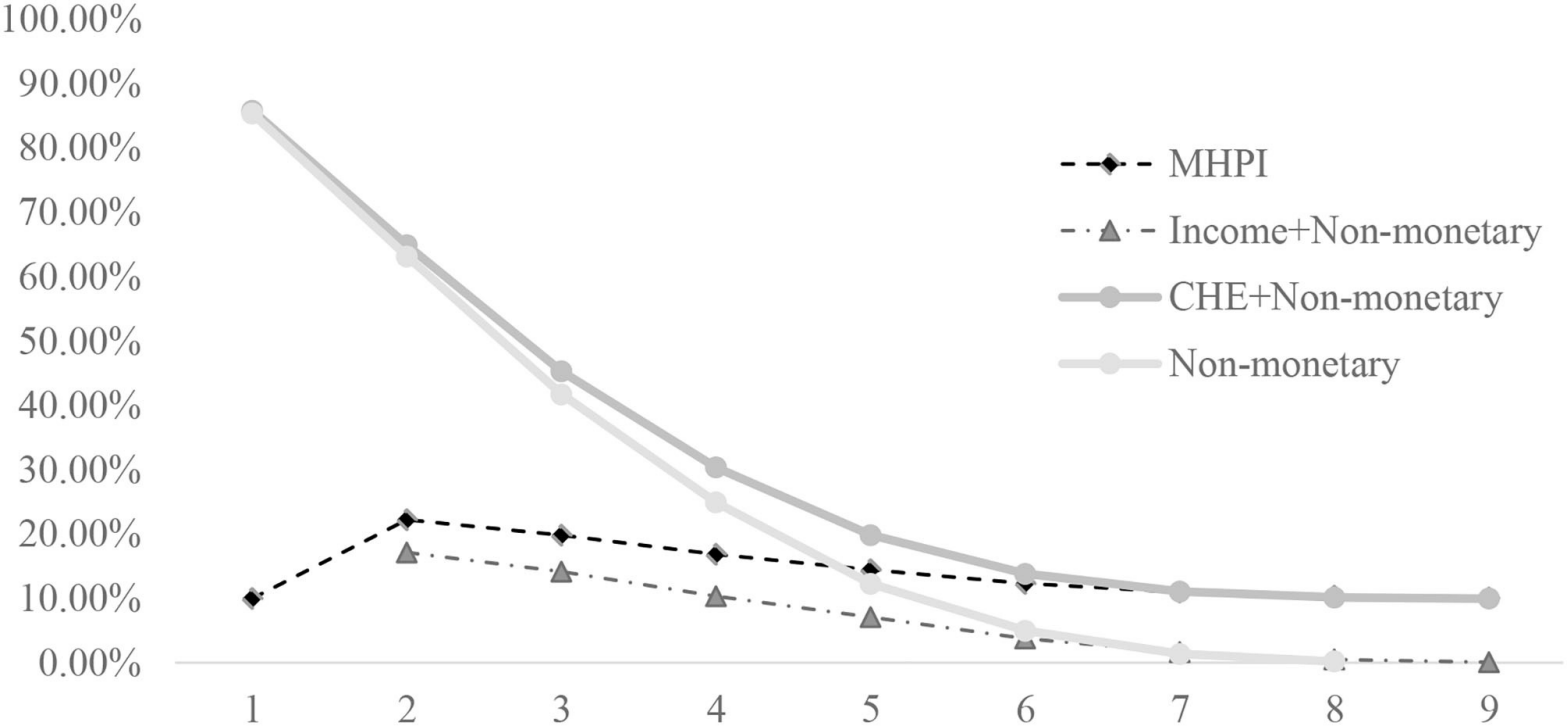
The comparison of *H* between MHPI, “Income + Non-monetary MHPI,” “CHE + Non-monetary MHPI,” and “Non-monetary MHPI.” The “Income + Non-monetary MHPI” is measured by taking relative income poverty as a necessary condition with eight non-monetary indicators in the A–F method. The “CHE + Non-monetary MHPI” is measured by taking CHE as a sufficient condition with 8 non-monetary indicators in the A-F method. The “Non-monetary MHPI” is measured only with 8 non-monetary indicators in the A–F method.

We further compare the MHPI with other health poverty indices calculated performing a different method. In Figure 1, we remove the CHE dimension to measure the “Income + Non-monetary MHPI” in the A-F method, the income dimension to measure the “CHE + Non-monetary MHPI,” and both monetary dimensions to measure the “Non-monetary MHPI.” First, we can observe that MHPI changes more smoothly than the other indices with increase in *k*, making it a smaller error and more universal than the other indices. Second, with increase in k, the gap between MHPI and “Income + Non-monetary MHPI” increases too, which indicates that households with higher dimensions of health deprivation are more likely to experience CHE. Third, when *k* ≤ 5, with the increase of *k*, the incidence of health poverty measured by “CHE + Non-monetary MHPI” and “Non-monetary MHPI” remains high and declines rapidly, which indicates that these two indices have little effect on the identification of health-poor households. When *k* ≥ 6, the incidence of multidimensional health poverty is relatively stable. Therefore, setting the cutoff *k* as 5 or 6 is of more practical value for investigating multidimensional health poverty.

In order to further investigate the differences between the method presented in this study and the traditional multidimensional health poverty identification method, we compare MHPI with the “Non-monetary MHPI.” When *k* = 5, “Non-monetary MHPI” means deprivation in at least five health dimensions, and when *k* = 6, MHPI also means deprivation in at least five health dimensions; besides, the incidence of multidimensional health poverty measured by the two indices is roughly the same. Therefore, we compare the “Non-monetary MHPI” at *k* = 5 with the MHPI at *k* = 6, and found that the number of healthy poor households calculated by MHPI is 1,292, and that number calculated by “Non-monetary MHPI” is 1,285, among which, 501 households in health poverty calculated by both methods. The overlap is only about 39%, indicating a large difference between the two measurements. Besides, 791 CHE households are not identified as health poor using the traditional multidimensional health poverty identification method, accounting for 76% of all households with CHE, and 893 households with relatively high income are identified as health poor in the traditional method, accounting for 69% of all health-poor households. This means that if we ignore monetary aspects, we will miss the vast majority of households who need financial help because of their health problems.

### The Decomposition of MHPI

To further understand the impact of each indicator on multidimensional health poverty, we measure the contribution of each indicator. In Table 5, CHE, relative income poverty, SRH, depression, cognitive impairment, and chronic diseases all contribute highly to health poverty at each cutoff, accounting for more than 70%. Taking *k* = 5 as an example, SRH accounts for the highest proportion and becomes the primary factor of health poverty, followed by CHE, relative income poverty, depression, cognitive impairment, and chronic diseases. The reason for this phenomenon may be that people with relatively low incomes are mostly engaged in manual labor. Long time and high-intensity physical labor can damage their physical health and cause them to suffer from diseases and incur greater medical expenses and heavy psychological burdens; whereas with the development of the economy of China, the household living standard has been significantly improved, and the proportion of people suffering from malnutrition is low. Improved medical care has led to a decline in the proportion of disabled people. The overall social atmosphere is harmonious, and the level of individual social health is high. Therefore, PADLs, nutrition, interpersonal relationships, and social trust contribute very little to multidimensional health poverty.

**Table 5:** Decomposition of MHPI under equal weights by all indicators.

**Dimension**	**Indicator**	***k* = 3**	***k* = 4**	***k* = 5**	***k* = 6**
Medical expenditure	CHE	10.80	11.97	13.51	15.77
Income	Relative income poverty	16.16	14.28	12.91	11.72
Physical health	SRH	13.48	13.73	13.83	13.61
	PADLs difficulties	7.37	7.65	7.76	8.09
	Chronic diseases	9.54	9.86	10.15	10.22
	Nutrition	5.42	5.46	5.43	5.40
Mental health	Depression	12.56	12.64	12.50	12.05
	Cognitive impairment	12.41	12.03	11.72	11.37
Social health	Interpersonal relationships	3.95	4.13	4.14	4.08
	Social trust	8.31	8.23	8.05	7.67

In terms of dimensions, the contribution rate of physical health to health poverty is about 37%, the contribution rate of monetary dimensions (CHE and relative income poverty) is about 26%, followed by mental health, which is 25%; the contribution rate of social health is the lowest, about 12%. It shows that physical and mental health, income, and medical expenditure are the most important dimensions affecting health poverty.

In addition, the contribution degree of each indicator is generally consistent under different cutoffs, indicating that the contribution degrees of health poverty indicators are relatively stable; that is, MHPI can comprehensively and clearly capture the dimensions affecting household health poverty.

### Urban-Rural Differences in Multidimensional Health Poverty

Considering the obvious gap in the economic development level, medical care, and education between urban and rural areas, we further conduct a comparative analysis between urban and rural areas from three aspects: incidence of multidimensional health poverty *H*, intensity of deprivation *A*, and multidimensional health poverty index *M*_0_and calculate their respective contribution shares.

In Table 6, the incidence of multidimensional poverty, intensity of deprivation, and MHPI in rural areas are all higher than those in urban areas at each cutoff. The contribution rate of rural households to MHPI is 20% higher than that of urban households, indicating that most multidimensional health poverty households are from rural areas. It is worth noting that when *k* = 1, that is, when only CHE is considered, the gap between rural and urban households is larger, and the contribution difference reaches 35%, indicating that rural households face a higher burden of medical expenditure than urban households. The reason may be that the reimbursement ratio of the New Rural Cooperative Medical Scheme (NRCMS) is less than that of Urban Employee Basic Medical Insurance (UE-BMI) and Urban Resident Basic Medical Insurance (UR-BMI), and most rural resident participants in the NRCMS. Moreover, the insurance consciousness of rural residents is weaker, and the purchase rate of commercial health insurance is less. The above reasons jointly result in a higher medical out-of-pocket rate in Chinese rural areas.

**Table 6:** Decomposition of MHPI by rural and urban.

***k***	***H%***		***A***		***M_**0**_***		**Proportion**	
	**Rural**	**Urban**	**Rural**	**Urban**	**Rural**	**Urban**	**Rural**	**Urban**
1	12.59	7.24	0.4988	0.4352	0.0628	0.0315	67.67	32.33
2	23.29	21.18	0.4606	0.4088	0.1073	0.0866	56.54	43.46
3	21.27	18.41	0.4853	0.4401	0.1032	0.0810	57.22	42.78
4	18.92	14.67	0.5084	0.4759	0.0962	0.0698	59.13	40.87
5	16.92	11.92	0.5212	0.4934	0.0882	0.0588	61.15	38.85
6	14.94	9.65	0.5240	0.4919	0.0783	0.0475	63.40	36.60
7	13.58	8.27	0.5164	0.4739	0.0701	0.0392	65.24	34.76
8	12.90	7.63	0.5068	0.4548	0.0654	0.0347	66.44	33.56
9	12.66	7.29	0.5012	0.4390	0.0635	0.0320	67.54	32.46

## Discussion and Conclusion

As noted above, health poverty is multidimensional and relevant to medical expenditure and income. When measuring health poverty, these factors should be integrated. As defined by the WHO, health includes physical health, mental health, and social health (31). Therefore, we select indicators from the three dimensions to measure the overall health status of the household. As for monetary factors, once CHE occurs, the household needs timely medical assistance. It means that health poverty occurs, so CHE should be regarded as a sufficient condition. Besides, the premise of health poverty is that health and poverty problems go hand in hand, that is, health problems lead to poverty or poverty leads to health problems, so income poverty should be regarded as a necessary condition for health poverty, which together with health problems constitutes health poverty. Based on the A-F method, our paper takes CHE as a sufficient condition and relative income poverty as a necessary condition and selects eight indicators from three non-monetary health dimensions, namely, physical health, mental health, and social health, to construct the MHPI.

First, the result shows that the incidence of health poverty measured by MHPI ranges from 10 to 22%, and varies significantly gently compared with the other indices. Therefore, when different cutoffs are used, the differences in health-poor households identified are smaller; that is, the recognition results are more stable. Second, there are significant differences between MHPI and traditional multidimensional health poverty methods without monetary aspects. The overlap of the two methods is small. The traditional method misses the majority of households suffering CHE and includes lots of households with relatively high incomes. Compared with the traditional method, the MHPI is more accurate and comprehensive. In summary, given the stability, accuracy, and comprehensiveness shown by MHPI, we believe that it is practicable to use MHPI to measure health poverty.

The decomposition measure of each indicator shows that physical health remains a major contributor to multidimensional health poverty, followed by economic conditions. Therefore, preventing the cycle of disease and poverty remains a top priority for health poverty alleviation. Besides, mental health accounts for a quarter of the total, so mental health issues are also a focus. The urban-rural decomposition shows that no matter how the cutoff is adjusted, the incidence of multidimensional health poverty in rural households is higher than that in urban households, and that high-dimensional health poverty is more likely to occur. In particular, rural households have a much higher incidence of CHE than urban households, indicating that rural households in China have a heavy burden of out-of-pocket medical payments, and that the reimbursement rate of rural medical insurance needs to be further improved.

There are still some limitations to be noted in this study. First, the cutoffs of cognitive impairment, interpersonal relationships, and social trust are chosen based on subjective social experience, lacking objectivity, and accuracy. Second, to reduce sample loss and maintain an adequate sample size, we retain households with missing multidimensional health indicators of a certain member, which may affect the accuracy of the results. Third, the study is based on cross-sectional data, but it would enrich the study if we use longitudinal data to study dynamic multidimensional health poverty. Future research can study the dynamic trend of dynamic multidimensional health poverty based on longitudinal data, or measure multidimensional health poverty in other countries for comparison. In addition, more studies are needed on the classification of multidimensional health poverty and its influencing factors, as well as corresponding health poverty alleviation measures.

In summary, the MHPI constructed in this study is consistent with the purpose of the health poverty alleviation policy of the government, and policymakers will find it useful in identifying multidimensional health poverty groups that truly need help. The setting of indicators in each dimension can be flexibly adjusted to assess health poverty in other countries according to the actual situation of each country and the availability of data. Our findings call for further improvements in medical care policies to minimize the economic risks posed by out-of-pocket medical payments for households and narrow the disparities between urban and rural areas in China. Moreover, focus on multidimensional health, especially mental health and social health, to achieve more effective and comprehensive health poverty alleviation strategies is needed.

## Author's Note

The measurement result evidence that physical health, monetary dimensions (CHE and income poverty), and mental health contribute most to health poverty in China. In addition, the MHPI is significantly higher in rural areas than urban due to higher out-of-pocket medical payments and health deprivation in more dimensions. Compared with the traditional method, the MHPI is more accurate, stable, and comprehensive, making it more suitable for measuring health poverty.

## Data Availability Statement

The original contributions presented in the study are included in the article/supplementary material, further inquiries can be directed to the corresponding author.

## Author Contributions

X-XC: conceptualization, methodology, and writing—original draft preparation. X-HL: formal analysis, writing—review and editing, and funding acquisition. Z-ZZ: data processing and formal analysis. All authors contributed to the article and approved the submitted version.

## Funding

This research was partly supported by the National Social Science Fund of China (18BGL200).

## Conflict of Interest

The authors declare that the research was conducted in the absence of any commercial or financial relationships that could be construed as a potential conflict of interest.

## Publisher's Note

All claims expressed in this article are solely those of the authors and do not necessarily represent those of their affiliated organizations, or those of the publisher, the editors and the reviewers. Any product that may be evaluated in this article, or claim that may be made by its manufacturer, is not guaranteed or endorsed by the publisher.

## Footnotes

^1^The criterion is from WHO: https://www.who.int/toolkits/growth-reference-data-for-5to19-years/application-tools
